# Phenotypic characterization of soybean genetic resources at multiple locations: breeding implications for enhancing environmental resilience, yield and protein content

**DOI:** 10.3389/fpls.2025.1422162

**Published:** 2025-04-07

**Authors:** Tomáš Vymyslický, Oldřich Trněný, Hendrik Rietman, Christiane Balko, Vuk Đorđević, Predrag Ranđelović, Magdaléna Dybová

**Affiliations:** ^1^ Agricultural Research, Ltd., Troubsko, Czechia; ^2^ Agro Seed Research bv, Opglabbeek, Belgium; ^3^ Julius Kühn-Institut, Federal Research Centre for Cultivated Plants, Institute for Breeding Research on Cultivated Plants, Sanitz, Germany; ^4^ Institute for Field and Vegetable Crops, Novi Sad, Serbia

**Keywords:** *Glycine max*, field trials, morphological and phenological traits, weather data, genotype-environment interactions

## Abstract

**Introduction:**

Soybean is an important legume crop and a leading source of dietary protein and oil in animal feed, as well as an important food for human consumption. The objective of our research was to study soybean genetic resources in context of future protein self-sufficiency both in human and animal nutrition.

**Methods:**

Collection of 360 different accessions from various regions worldwide was evaluated across four European locations during two consecutive years in phenotyping trials. The five most important traits of soybean – plant emergence, plant length, protein content, seed yield, and R8 stage – were carefully analysed, revealing significant variability.

**Results:**

Ten exceptionally stable genotypes were identified based on their protein content and yield, presenting promising candidates for breeding programs.

**Discussion:**

Our findings underscore the importance of integrating genotype-environment interaction analyses into breeding initiatives, considering the observed variability in phenotypic traits across diverse environments and genotypes.

## Introduction

Plant Genetic Resources for Food and Agriculture are defined as diversity of genetic material contained in traditional varieties and modern cultivars, as well as crop wild relatives and other wild plant species that can be used now or in the future for food and agriculture (Halewood et al., 2018). Preservation of biodiversity and natural variation within species has become a global concern. The diversity within a species allows it to survive and adapt to new environments, new pests, and changing climates (Iriondo et al., 2008). Genetic resources are essential for maintaining and enhancing the efficiency and the resilience of production systems, as well as for healthy diets and the delivery of ecosystem services, such as pollination and pest and disease regulation (Halewood et al., 2018). Genetic resources are essential sources of genes for breeding. In an environmentally dynamic world, with constantly increasing population and limited resources, we need to conserve genetic diversity for our own food and environmental security (Iriondo et al., 2008). Agricultural production is dependent on genetic resources domesticated elsewhere and subsequently developed in other countries and regions. The erosion of these resources poses a severe threat to the world’s food security in the long term (Ebert, 2020). The importance of genetic resources has also been increasing recently due to ongoing climate change. Crop breeders and their growers are increasingly forced to face climatic extremes precisely with the use of promising genetic resources in breeding processes (Muluneh, 2021).

Modern cultivated soybean was domesticated from the annual wild soybean (*G. soja* Sieb. & Zucc.) in East Asia 6000–9000 years ago (Kim et al., 2012). The distribution of wild soybean is limited to East Asia – China, Japan, Korea and part of Russia (Jeong et al., 2019). Within this region, China is considered to be the domestication centre of soybean. China has the earliest written records of soybean cultivation (Kim et al., 2012). Soybean has been found in unearthed artefacts across dynasties and provinces (Qiu and Chang, 2010). The crop was introduced to Europe in the 18^th^ century and then to the United States (Hymowitz and Harlan, 1983). The five countries with the highest soybean production in the world are Brazil, United States, Argentina, China and India (FAOSTAT, 2023).

Soybean (*Glycine max* (L.) Merr.) is an important legume crop and a leading source of dietary protein and oil in animal feed, as well as a staple food for human consumption (Hartman et al., 2011). Soy protein is one of the major components of the livestock diet and it is increasingly important in the human diet. Soybean is a rich source of high-quality proteins containing all the essential amino acids found in animal proteins (Gorissen and Witard, 2018). Soybean seeds contain approximately 35 - 40% of proteins, 20% of lipids, 9% of dietary fibre and 8% of moisture (He and Chen, 2013). The content of starch in the seeds is about 6%, total soluble sugars is about 9% and sucrose about 7% (Sharma et al., 2014). Soybean and its products are rich sources of minor non-nutrient components with potential health benefits (Cicero et al., 2017), which are often referred to in the literature as phytochemicals (biologically active proteins and peptides, lectins, lunasin). Soybean products are important sources of isoflavones, phytosterols, phytic acid and saponins (Barnes, 2010). Because of high content of polyunsaturated fats, fibre, vitamins and minerals and low content of saturated fat, soybean products have many beneficial health and therapeutic effects (Zuo et al., 2023; Chen et al., 2019; Fan et al., 2022; Kao and Chen, 2006; Liu et al., 2005). Through fermentation by *Bacillus subtilis* or *Aspergillus oryzeae*, the nutritional value of fermented soybean products is enhanced. Fermentation improves the digestibility, and increases the soy protein and isoflavone profiles compared to non-fermented soy foods (Jayachandran and Xu, 2019).

The biggest commercial interest in soy is the protein and oil. In soybean, the protein content is negatively correlated with the oil content and the yield (Ohara and Shimamoto, 2002). Soybean protein is the only plant protein source, containing all the essential amino acids necessary for humans. Soybean protein does not contain cholesterol, and the contents of methionine and branched-chain amino acids are low compared with animal proteins (Gorissen and Witard, 2018).

Genetic resources play very important role in improving and breeding of all agricultural crops. In soybean, genetic resources play an important role in increasing the resistance and tolerance to abiotic and biotic stressors. The main aim of current breeding efforts is promoting the sustainable production of soybean worldwide. Resistant or tolerant genetic resources could be used as input materials in the forthcoming selection of soybean varieties with high protein content and high yield (Guo et al., 2022).

Soybean is currently the most important protein crop in Europe (Guilpart et al., 2022). EU soybean production is 2.7 million tons, while non-EU European countries produced another 8.4 million tons (EUROStat, 2021; FAOStat, 2021). European production covers only a small part of soybean demands. The European Union imported an annual average of 14 million tons of soybeans and 18 million tons of soybean cake (EUROStat, 2021). Most of the import is from America (USA, Argentina, Brasil). The high demand for soybean protein in Europe is therefore an important reason for expanding soybean cultivation to central and northern growing areas where it is a minor crop. European self-sufficiency in soybean would require 9 – 12% of its arable land to be sown to this crop (Guilpart et al., 2022). High yielding and resistant varieties of soybean, with stable yields in different environmental conditions and stable protein contents, are the basis for the future protein self-sufficiency of Europe (Gorissen and Witard, 2018).

Within the EUCLEG project “Breeding forage and grain legumes to increase EU’s and China’s protein self-sufficiency” (www.eucleg.eu) promising genetic resources from different regions in the world were gathered and subjected to detailed phenotyping under the conditions of four different localities in Europe.

In order to provide a solid foundation for the utilization of soybean genetic resources in breeding programs, we aimed at (i) identification of promising soybean accessions for future breeding, (ii) characterizing the phenotypic diversity of agriculturally relevant traits at multiple locations and (iii) getting an insight into the extent of accession × location interaction for future adaptive breeding efforts. Based on the evaluation, the most promising materials were proposed for the further use in breeding process and discussed in the context of global climate change.

## Materials and methods

### Soybean genetic resources

A set of soybean genetic resources was gathered from the EUCLEG consortium members, breeders, research institutes and gene banks. Accessions were delivered with specific material transfer agreements from each supplier to a central coordinator, who further distributed seeds under the same agreements to the researchers performing the experiments. Altogether 360 accessions of different origin were evaluated in the trials. Most accessions originated from Europe (242 accessions), North America (44 accessions) and Asia (30 accessions). Some accessions had unknown origin (44 accessions). The most represented countries were Belgium (50 accessions), Serbia (38 accessions), Germany and Canada (33 accessions) and China (21 accessions).

### Experimental sites

Four different localities of soybean trials representing different agroclimatic regions of Europe were chosen: Troubsko – Czech Republic (CZE), Novi Sad – Serbia (SRB), Groß Lüsewitz – Germany (GER) and Kessenich – Belgium (BEL). Localities Troubsko and Novi Sad belong to Pannonian region, Kessenich belongs to North Maritime region and the locality Groß Lüsewitz belongs to Continental zone (Ceglar et al., 2019).

The same trials were conducted both in 2018 and in 2019. The plot size used in the trials was 5 m^2^. Sowing density was 75 seeds/m^2^ for maturity group MG 000, 65 seeds/m^2^ for the MG 00, 55 seeds/m^2^ for the MG 0 and 45 seeds/m^2^ for the MG I/II. Information about maturity groups was obtained from the breeders or sales representatives of the variety. Plants for the evaluation were taken from the inner part of the plot. Irrigation was only applied at the Kessenich location in 2018. The amount of water used for irrigation was included in the rainfall totals shown in **Table 1**. Herbicide treatment was applied if necessary. Fertilisation was applied in each year before sowing using the following quantities: N – P – K 45 – 45 – 45 kg.ha^-1^.

**Table 1:** Layout of field trials at the different locations.

Parameter	Trial location							
	BEL	BEL	GER	GER	CZE	CZE	SRB	SRB
	2018	2019	2018	2019	2018	2019	2018	2019
Number of accessions	360	360	100	100	100	100	360	360
– with 1 plot	320	320	76	76	76	76	320	320
– with 2 plots	32	32	16	16	16	16	32	32
– with 4 plots	8	8	8	8	8	8	8	8
Number of plots	432	432	140	140	140	140	432	432
Plot size	1 x 5 m	1 x 5 m	1,5 x 3,35 m	1,5 x 3,35 m	1.25 x 4 m	1.25 x 4 m	2 x 4 m	2 x 4 m
Distance between rows	0.25 m	0.25 m	0.25 m	0.25 m	0.25 m	0.25 m	0.5 m	0.5 m
Sowing date	7. 5.	10. 5.	3. 5.	7. 5.	3. 5.	16. 4.	19. 4.	7. 4.
Inoculum	Yes	Yes	Yes	Yes	Yes	Yes	No	No

### Experimental design

A partially replicated plot design was used for all the localities, see **Table 1**. GER and CZE locality had 100 soybean accessions and BEL and SRB locality had 360 accessions, in one, two or four replications. Border plots were used in order to avoid the marginal effect.

For the inoculation the product NITRAZON was used (Farma Žiro, Ltd. Czech Republic). In all locations except Novi Sad, soybean seeds were inoculated before sowing. In Novi Sad the inoculation was not necessary, due to the long-term cultivation of soybeans and its representation in crop rotations. Therefore, there was a sufficient supply of *Rhizobium* bacteria in the soil at this location.

We used different seeding densities for each maturity group, because varieties with a later ripening period have a longer growing season and the plants reach larger size. A detailed description of this collection is provided in **Supplementary Table 1**.

### Evaluated morphological and phenological data

The traits listed in **Table 2** were evaluated. Phenological stages were evaluated using the BBCH scale for soybean (Munger et al., 1997). The protein content, was uniformly assessed for all genotypes at the EV-ILVO institute in Belgium employing NIRS (Near Infra Red Spectrometry), using the methodology described by Zhu et al., 2018.

**Table 2:** Overview of evaluated morphological and phenological traits.

No.	Observation	Score	Remarks
1	V-stage at emergence	None – VE – VC	BBCH scale for soybean (Munger et al., 1997).
2	Plant emergence	1-9	1 low, 9 high
3	Plant vigour at emergence	1-5	1 low, 5 high
4	V2 stage	date	Date when 50% of the plants of a plot are in V2. BBCH scale for soybean (Munger et al., 1997).
5	Plant height at V2	cm	BBCH scale for soybean (Munger et al., 1997).
6	R1 stage	date	Days from sowing. BBCH scale for soybean (Munger et al., 1997).
7	R2 stage	date	Days from sowing. BBCH scale for soybean (Munger et al., 1997).
8	Diseases, pests	1-9	1 absence, 9 strong attack
9	Abiotic stress	1-9	1 no stress, 9 severe stress
10	R8 stage	date	Days from sowing. BBCH scale for soybean (Munger et al., 1997).
11	Lodging at R8	1-5	1 no lodging, 5 abundant lodging. BBCH scale for soybean (Munger et al., 1997).
12	Seed yield	kg.ha^-1^	Expressed at 14% moisture content. Seed yield was calculated in kg.ha^-1^ from each experimental plot.
13	Moisture content	%	Drying for 72 hours at 70°C
14	Protein content	%/dry matter	Analysed by NIRS
15	Seed weight	g	4 x 50 seeds (expressed at 14% moisture content)
16	Plant length	cm	5 plants/plot
17	Height first pod	cm	5 plants/plot
18	Mottled seeds	%	From the dried sample
19	Node number on the main stem of the plant	counting	5 plants per plot
20	Number of branches with pods	counting	5 plants per plot
21	Distribution of pods on the plant	1-5	5 plants per plot, 1 - pods only in the basal part, 5 - pods distributed evenly on the plant
22	Seed number	counting	5 plants per plot (after threshing)
23	Seed weight per plant	g	5 plants per plot (after threshing) calculated

### Data analyses

To analyze the five traits considered to be the most important (plant emergence, plant length, date of reaching the R8 stage, seed yield and protein content), we employed a combination of statistical approaches. First, we built a linear mixed model, from which we performed variance component analysis and derived BLUPs (Best Linear Unbiased Predictions). Next, we conducted principal component analysis (PCA) to explore the structure phenotypic expression of genotypes, followed by redundancy analysis (RDA) to assess the influence of genetic structure on trait variation. Finally, we used Bayesian regression to analyze genotype-environment interactions (GxE).

We evaluated traits results using a linear mixed models to account for both fixed and random sources of variation. The results for the selected traits were used as the predicted variables to set up the models. Based on these models, we assessed variance components to investigate the influence of genotype on these five plant traits in deferent trials. The mixed model analysis was conducted using the R package “lme4” (Bates et al., 2015). The seed yield data were standardized to z-scores by subtracting the mean of each variable and dividing by its standard deviation, which transforms the data to have a mean of 0 and a standard deviation of 1 to enhance the stability of model parameter estimation. Each trial assignment was used as an independent fixed effect in the model. Random effects were included to account for genetic variability of genotypes and to control for the influence of row and column positions within each trial. Geographic location, season, and their interaction were not considered due to the significant variation in environmental conditions across both years. Statistical significance of random and fixed effects was assessed using backward elimination procedure implemented in lmerTest R package (Kuznetsova et al., 2017). The step function testing random effects with likelihood ratio tests and fixed effects with F-tests using Satterthwaite’s method for degrees of freedom. Weather characteristics were initially included as fixed effects but were removed from the final model due to non-significance during the model selection procedure. Convergence of model estimates was verified using diagnostic plots, including residuals versus fitted values and quantile-quantile plots, to ensure the assumptions of normality and homoscedasticity were met. To estimate the marginal means of the trials with standard errors (SE) and 95% confidence intervals (CI), we used the *emmeans* package in R (Lenth, 2024). Generalized heritability (Cullis et al., 2006) was computed based on genotypic variance and BLUP conditional variance, appropriate for linear mixed models in partially replicated and unbalanced plot designs.

Formula 1: Formula of used linear mixed models to predict and analyse selected traits


$$y_{ijkl}=\;\beta_{0}+\;\beta_{i}Trial_{i}+\;u_{j}+\;v_{ik}+\;w_{il}+\;\epsilon_{ijkl}$$

In this model, *y_ijkl_* represents the outcome for a given trait, with *β*_0_ as the intercept. The fixed effect *β_i_* Trial_i_ captures differences associated with the *i^th^* trial. Random effects include *u_j_* representing genetic variation due to genotype *j*, *v_ik_* which accounts for variability across the *k^th^* row within the *i^th^* trial, and *w_il_* representing the effect of the *l^th^* column within the *i^th^* trial. Finally, *ϵ_ijkl_* is the residual error term.

BLUPs were derived from the mixed models to estimate the genetic performance of each genotype, accounting for other effects and sources of variability included in the models. Combined correlation plots of BLUPs (Best linear unbiased prediction) were generated utilizing the Performance Analytics R package. BLUPs are used in linear mixed models for the estimation of random effects. Pearson correlation coefficients and the statistical significance tests among variables were computed using the cor.test function from the stats package. Principal component analysis (PCA) was conducted using prcomp function from R Stats 4.0.3 package on unit variance scaled BLUPs in R. PCAs was plotted using fviz_pca package.

Redundancy Analysis (RDA) was conducted to quantify the influence of genetic groups on phenotypic traits. The genetic assignment based on genotyping using the 355K SoySNP microarray (Wang et al., 2016) from Saleem et al. (2021) was used to categorize analysed genotypes into five distinct genetic groups and an admixed group. RDA was conducted using the Vegan 2.5-7 R package, utilizing Z-score scaled BLUPs of R8, protein content, plant length, plant emergence, and seed yield traits. Genetic cluster assignments were employed as constrained variables. The biplot function was used to generate the plot, with scaling for the genetic groups (scaling = 2).

The stability of genotypic performance across our multi-environment trials was assessed utilizing a Bayesian Finlay-Wilkinson regression framework, as implemented in the FW package for R (Lian and de los Campos, 2016). This approach allows for the nuanced characterization of genotype-environment interactions by estimating model parameters that reflect genotypic performance in response to environmental conditions. The analysis integrated a covariance matrix to adjust for genotypic similarities among accessions, using a marker-derived kinship matrix, and considered environmental variability among trials using a covariance matrix of scaled climatic data (**Table 1**). Bayesian estimations of environmental and genotype model parameters were conducted using the GibbsA method, specified in the FW package with the following settings: 1,000,000 iterations for thorough exploration of the parameter space, a burn-in period of 100,000 to ensure model stabilization, and a thinning interval of 10,000 to reduce autocorrelation in the sampled parameters.

## Results

### Weather data

Weather data from all the experimental sites were collected and are presented in **Table 3**. From the presented data the clear difference between the two with continental climate (Novi Sad and Troubsko) and two with oceanic climate (Kessenich and Groß Lüsewitz) can be seen. The difference is clearly visible in the temperatures, average temperature of growing season (1.4.-31.10.) varied from 14,2°C (Groß Lüsewitz, 2019) to 20,1°C (Novi Sad, 2018). The sum of effective temperatures above 10°C varied from 2048 (Groß Lüsewitz, 2019) to 3490 (Novi Sad, 2018). The day of the last frost from 1. 4. varied from 0 (more localities) to 44 days (Groß Lüsewitz, 2019). The day of the first frost before 1. 11. varied from 0 (more localities) to 26 (Groß Lüsewitz, 2019). Based on temperatures, Novi Sad was the warmest location, while Groß Lüsewitz was the coldest. Precipitation was distributed rather randomly, with significant drought recorded in 2018 in the localities Troubsko and Kessenich. If we focus on precipitation, two parameters are shown in the **Table 2** – sum of precipitation in the period 1. 4. – 31. 10. (soybean growing season) and sum of precipitation in the period 1. 4. – 31. 5. (emergence period). While in the whole growing period the driest was the locality Kessenich (188 mm), in the emergence period the driest was the locality Troubsko (47 mm). The driest locality was Kessenich in 2018, while the wettest location was Novi Sad in 2019. In 2018, only the Kessenich site received irrigation. However, temperatures and precipitation need to be considered together, because higher temperatures mean higher evaporation.

**Table 3:** The most important weather data of all the experimental sites.

Country	BEL		CZE		GER		SRB	
Locality	Kessenich		Troubsko		Groß Lüsewitz		Novi Sad	
Coordinates	51°08’N, 5°48’Е		49°10’N, 16°30’Е		54°07′N, 12°32’E		45°20’N, 19°51’Е	
Year	2018	2019	2018	2019	2018	2019	2018	2019
Average temperature in the growing season (1. 4. - 31. 10.)	16.8°C	15.6°C	17.6°C	15.8°C	15.3°C	14.2°C	20.1°C	19°C
Sum of effective temperatures above 10°C during the growing season (1. 4. - 31. 10.)	2741	2364	3066	2621	2379	2048	3490	3281
Average temperature in the summer full growing season (1. 7. - 31. 8.)	20.6°C	19.3°C	22.2°C	20.6°C	19.2°C	18.1°C	23.2°C	23.9°C
Day of last frost after 1. 4.	0	13	6	37	33	44	0	0
Day of first frost before 1. 11.	0	0	0	24	13	26	0	0
Sum of precipitation in the growing season (1. 4. - 31. 10.)	188 mm	294 mm	238 mm	380 mm	263 mm	417 mm	226 mm	439 mm
Sum of precipitation in the emergence period (1. 4. - 31. 5.)	72 mm	55 mm	47 mm	95 mm	75 mm	57 mm	113 mm	202 mm
Sum of precipitation in the summer full growing season (1. 7. - 31. 8.)	51 mm	64 mm	55 mm	116 mm	80 mm	135 mm	78 mm	100 mm

### Evaluated traits and their variability

The summarising analysis of plant emergence, plant length, protein content, R8 stage, and seed yield as delineated in **Table 4** offers insights into the variability of these traits across different locations and growing seasons. Original measured values are in **Supplementary Table 2**. The trial conducted in Kessenich during the 2019 growing season showcased the most favourable outcomes. This particular trial is indicative of outcomes approaching ideal cultivation conditions, thereby elucidating the genotypes’ yield potential in the absence of abiotic stressors. Conversely, the 2018 Troubsko trial exhibited the least favourable results, a consequence of several adverse factors, most notably a deficiency in moisture during the initial stages of plant emergence followed by an excessively dry season. Despite these unfavourable conditions, the results from the Troubsko trial are invaluable for identifying genotypes with exceptional resilience, capable of contributing positively under less-than-ideal circumstances. A notable disparity was observed between the highest yielding trial and the least productive trial. The intermediate trials have conspicuously demonstrated the genotypes’ potential under the standard growth conditions prevalent at the respective locations.

**Table 4:** Summary of mean values and standard deviations (SD) of raw measured trait values across all trials.

		Plant emergence class	Plant length [cm]	Seed protein content [%]	R8 phase	Seed yield [kg.ha^-1^]
Groß Lüsewitz	2018	6.3 (1.5)	58.2 (17.2)	40.1 (1.8)	118.4 (25.2)	2760 (855)
*GER*	2019	6.3 (1.2)	71.2 (16.1)	40.7 (2.1)	133.4 (17.1)	2902 (594)
Novi Sad	2018	7.2 (1.4)	63.7 (20.9)	42 (1.7)	112.2 (14.2)	2414 (833)
*SRB*	2019	8.9 (0.7)	66.1 (19.2)	42.7 (2.1)	132.2 (12.8)	2244 (800)
Kessenich	2018	7.7 (2)	54.3 (11.5)	37.9 (3.1)	121.1 (14.5)	2761 (851)
*BEL*	2019	9.5 (1.1)	98.7 (20.9)	37.9 (2.2)	137 (16.6)	4204 (1311)
Troubsko	2018	5.7 (0.8)	42.9 (6.4)	44.5 (1.6)	166.8 (12.9)	385 (235)
*CZE*	2019	8 (0.7)	64.8 (12.4)	41.3 (2.6)	167.2 (16.7)	1320 (612)

SD are given in parentheses. R8 are days from sowing to the maturity stage.

A significant variation in protein content was observed across genotypes, years, and locations. The most notable values were recorded at Troubsko and Novi Sad, with figures reaching up to 50%. The average protein content values derived from the trials ranged between 38% and 45%. Trials conducted within the same locality yielded similar mean values. For detailed results, see **Table 4**.

**Figure 1:**
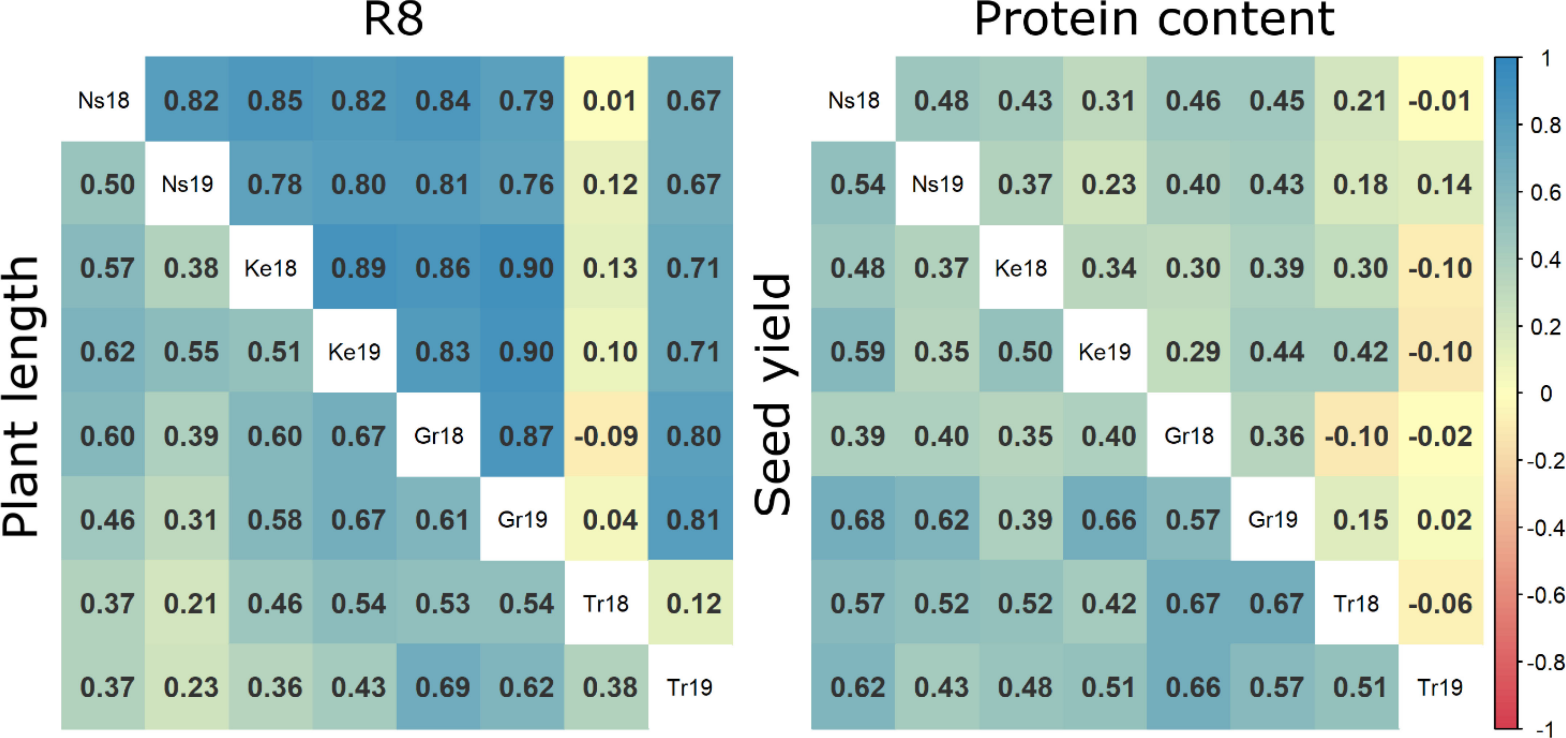
Comparative heatmaps illustrating Pearson correlation coefficients for measured data of plant length, R8, seed yield and protein content across various crop trials. Ns18 = Novi Sad 2018, Ns19 = Novi Sad 2019, Ke18 = Kessenich 2018, Ke19 = Kessenich 2019, Gr18 = Groß Lüsewitz 2018, Gr19 = Groß Lüsewitz 2019, Tr18 = Troubsko 2018, Tr19 = Troubsko 2019.

The correlation analysis of R8 stage, protein content, and seed yield (**Figure 1**) across different trials elucidates the general relationship among these trials. Higher correlation values suggest a diminished influence of genotype-environment interactions on trait values. Conversely, lower correlations between trials indicate variability in genotype responses due to environmental influences within each trial. The R8 stage trait exhibited the most consistent positive correlation across trials, except for those from the Troubsko 2018 trial, showing a predominantly strong correlation. For plant length and seed yield, medium to strong and consistent correlations were observed across all trials, including those under extreme conditions, without any exceptions. In contrast, protein content outcomes displayed the weakest correlation among trials. While the majority of trials exhibited weak to moderate positive correlations, the results from the Troubsko 2019 trial showed no discernible correlation with those of other trials for protein content.

### Linear mixed models

To analyze the individual factors and variance components influencing the results of the multi-trial experiment, we fitted a linear mixed regression model. From this model, we estimated the effects of the trials and calculated the Best Linear Unbiased Predictor (BLUP) (**Supplementary Table 3**) for four key soybean traits: seed yield, R8 stage, protein content, and plant height. Summary characteristics of the fitted models are provided in **Supplementary Table 4**.

The linear mixed regression models for plant length, protein content, R8 stage, and seed yield revealed statistically significant effects (α < 0.01) attributed to all fixed and random effects included in the models. The maximum likelihood estimation process successfully converged for each trait’s mixed linear model, indicating that was found stable and optimal estimates for both fixed effects and variance components. Variance components showed substantial contributions from different sources, including genotype, position in trial and residuals. Furthermore, the genotype variance component was higher than the variance components of random effects originating from plot rows and columns. The fixed effects on the traits showed notable variation across trials, reflecting primarily the differences in environmental conditions. All models exhibit residuals with a mean close to zero and normal distribution, indicating that the models effectively captured the variation.

Environmental conditions significantly modulated crop performance across trial sites. Statistical testing of fixed effects revealed a significant trial-dependent effect on all four traits. The Troubsko 2018 trial, characterized by extreme dry conditions, demonstrated the most negative impact on plant length and seed yield while simultaneously exhibiting the highest protein content. Conversely, the Kessenich trials (2018 and 2019) represented optimal growth conditions, showing the most substantial positive effects on seed yield, with Kessenich 2019 additionally displaying the most significant increase in plant length. Maturity phases (R8) varied across locations, ranging from 112 to 163 days, with Czech Republic sites consistently presenting the longest maturity period at 163 days.

Generalized heritability (Cullis et al., 2006) was highest for the R8 stage (92.1%), followed by protein content (84.7%) and plant length (84.5%). The lowest heritability 70.3% was observed for seed yield.

**Figure 2:**
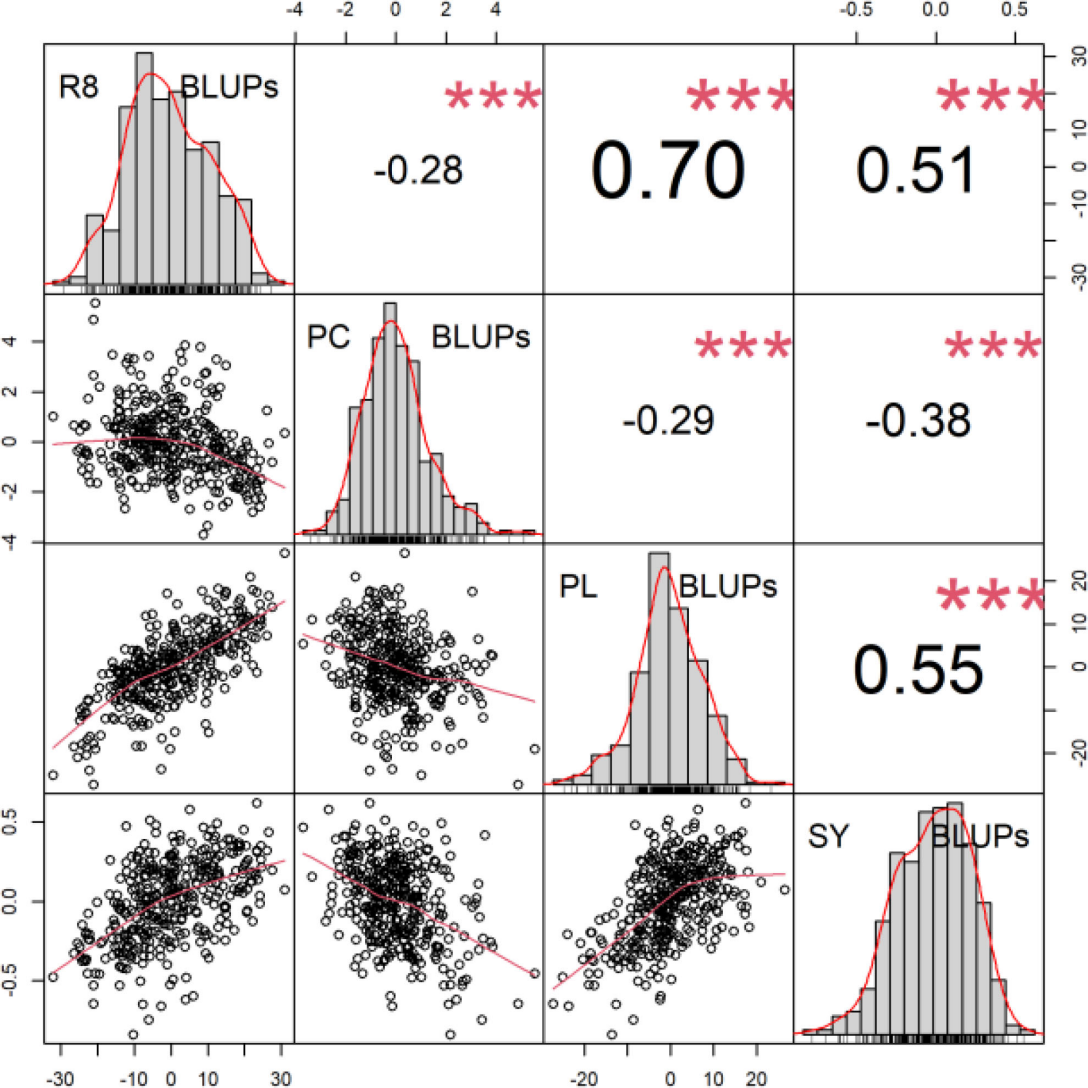
Correlogram for BLUPs of R8, protein content (PC), plant length (PL) and seed yield (SY). Scatter plots of relationship between parameters are described below diagonal. The correlation coefficient and the results of the cor.test are displayed above the diagonal. The stars mark the significance level of the test for association between paired samples using Pearson’s product moment correlation coefficient α *** < 0.01.

**Figure 3:**
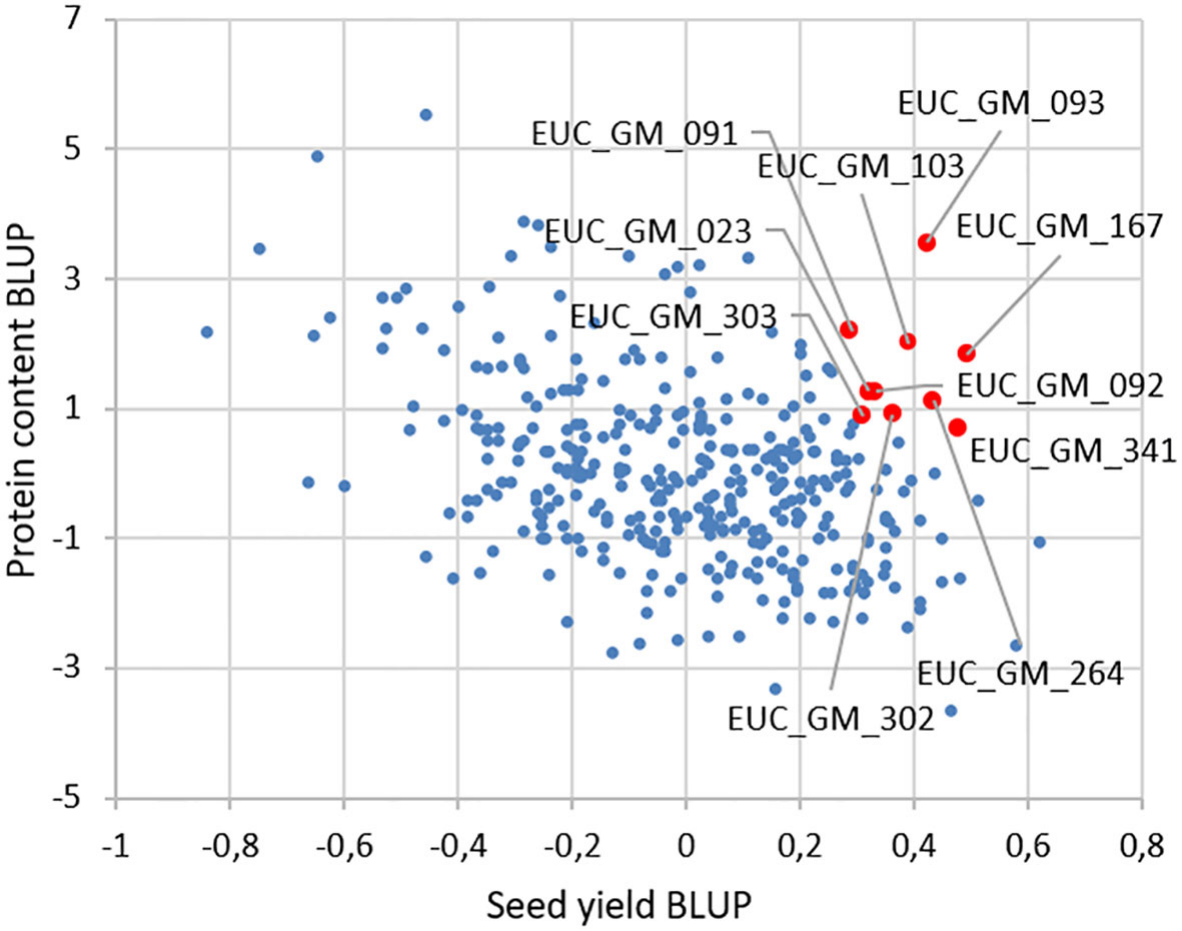
BLUP values for protein content and seed yield from all the soybean trials. By the red dots are marked the top ten soybean genotypes concerning the protein content and the seed yield.

Correlations of BLUP´s are presented in **Figure 2**. The most significant correlation was found between the plant length and the date of R8 (maturity) phase observation. The second strongest correlation was between the plant length and the seed yield. The third strongest positive correlation was between the seed yield and the date of R8 (maturity) phase observation. A notable inverse relationship was observed between protein content and other analyzed traits. The results of the BLUPs for protein content and seed yield are presented in **Figure 3**. The ten most promising genotypes are marked in the graph by the red colour and the accession name.

### Multivariate analysis

**Figure 4:**
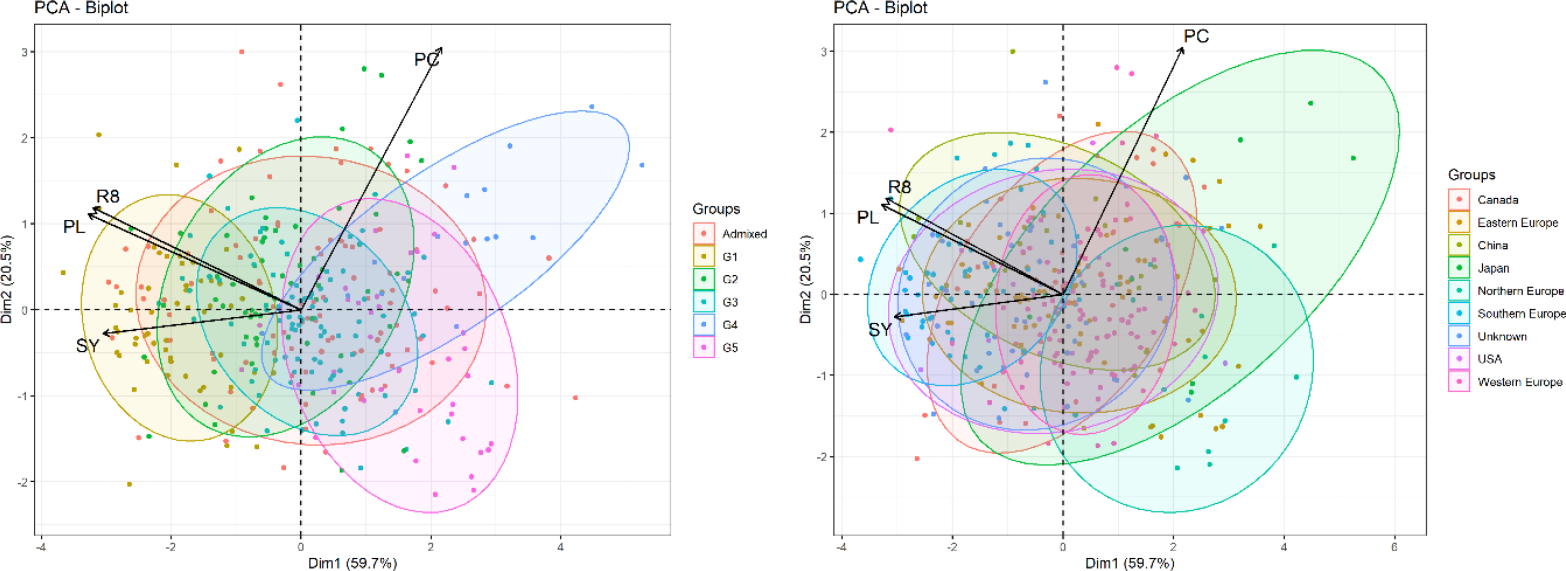
Comparative PCA plots of BLUPs of R8 stage, plant length, protein content and seed yield and distribution by genetic groups and the origin of accessions. The left plot categorizes observations into six groups (G1-G5, Admixed) and the right plot by geographic origin. Arrows show loadings of variables and ellipses show distribution of the accession origin.

We conducted a multidimensional reduction of BLUPs for plant length, R8 stage, protein content, and seed yield using PCA analysis. The results were then compared with the assignment of five genetic groups (Saleem et al., 2021) and regional origin of accession to assess their distribution across the derived principal components. PCA plots (**Figure 4**) illustrate the spread and overlap of data points within the first two principal components, explaining 59.7% and 20.5% of the variance, respectively.

PCA plots, annotated with accession assignments, show an overview of the relationships between the profiles of key traits of accessions and their genetic groupings or origins. he distribution of accessions according to their phenotypic profiles of key traits partially reflects patterns in genetic groups and origins, but many of them are tightly overlapped. Genetic groups and origins are interconnected because the positions of some groups reflect their origins.

Redundancy Analysis (RDA) quantifies potential trait associations and genetic diversity within the soybean population. RDA assesses the relationship between the predicted breeding values (BLUPs) of plant length, protein content, seed yield and R8 stage traits and five genetic groups (G1 to G5). The analysis aimed to discern how genetic groupings might explain variation in trait BLUPs. The partitioning of variance in our RDA model revealed that the genetic groups accounted for 40% of the variance in trait BLUPs, according to the adjusted R-squared value. This indicates a considerable effect of genetic background on the expression of the traits we evaluated. Examining the eigenvalues and their contribution to the total variance, we found that the first RDA axis (RDA1) explained a substantial proportion of the variance at 35% with an eigenvalue of 1.4. The subsequent axes (RDA2 to RDA4) explained smaller fractions of the variance, specifically 8.94% for RDA2, 5.9% for RDA3, and 0.9% for RDA4. The analysis highlighted the significance of the association between trait BLUPs and genetic groups. The traits scores provided insight into how specific trait BLUPs respond to the genetic grouping. The strongest response on RDA1 shows trait R8 stage followed with seed yield and plant length. Conversely, the BLUP for trait protein content had the lowest association with RDA1, which represents 88% of genetic grouping influence on traits. In summary, the RDA has shown that genetic background, as represented by genetic groups G1 to G5, is a significant predictor of trait variation. This relationship is crucial for our understanding of the genetic architecture of these traits and could have implications for selective breeding programs. Graphic inspection of the results (**Supplementary Figure 1**) suggests that membership to genetic group G1, in particular, have a strong positive influence on the traits R8 stage, plant length and seed yield.

### Genotype by environment interaction

**Figure 5:**
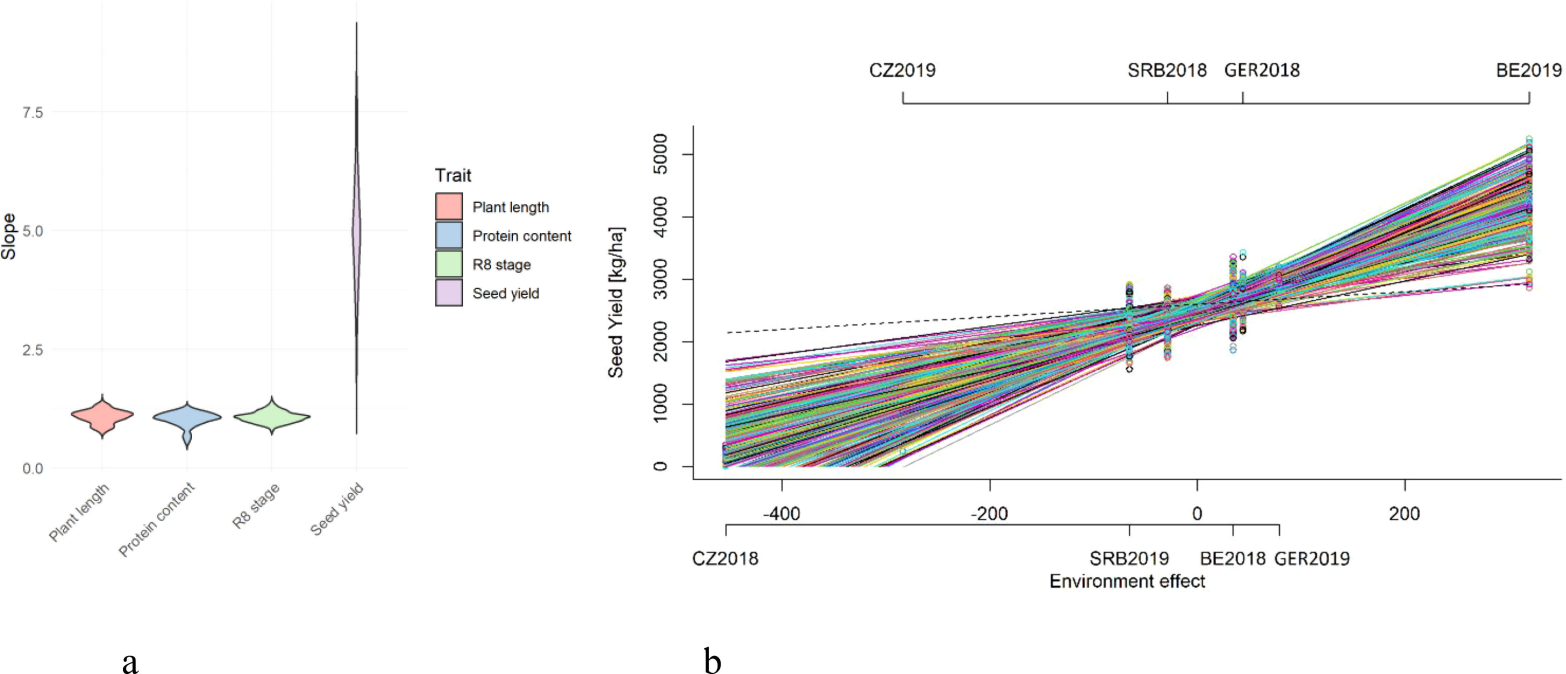
Distribution of Finlay-Wilkinson regression slope values for selected traits **(a)**. Genotypic response to estimated environmental variation in seed yield across eight distinct trials **(b)**. Each line represents the performance of genotype and the slope of line represents stability of an accession across different trial environments. Dashed line corresponds to a slope equal to b=1.

The stability of genotypic performance across diverse environments was evaluated using the Finlay-Wilkinson regression method. This approach measures genotype responses to environmental variability. Trait results for the R8 stage, plant length, protein content, and seed yield of each genotype were modelled against an environmental score, which denotes the average trait values for all genotypes within each environment. The regression line’s slope for each genotype acts as a stability indicator. Environmental scores were calculated for each trial, and Bayesian methods were employed to derive the slope values for each genotype. Our analysis disclosed a spectrum of stability responses across the genotypes for different traits (**Figure 5a**). Protein content displayed the most substantial stability. However, protein content also exhibited various responses to environmental changes, allowing us to distinguish between stable and responsive genotypes. In contrast, seed yield is exclusively associated with a positive response to an improving environment (**Figure 5b**) as a result of extreme environmental influences across trials, in which case, when the slope is far above 1, we have to compare genotypes relative to each other, with more stable seed yield genotypes being exhibited by individuals with lower slope values. Genotypes with a slope value near 1 were response stable. A slope of 1 indicates that the genotype’s trait values increase proportionally with improvements in environmental conditions. Those genotypes are considered to have a stable response to environmental changes and are generally stable across environments. Conversely, genotypes with the values of the slope significantly greater than 1 showed positive response. These genotypes are more responsive to environments. Such genotypes perform exceptionally well in good conditions but may suffer more in poorer conditions. Although all genotypes have a slope for seed yield greater than 1 (**Figure 5b**), for other traits (**Figure 5a**), genotypes with slope less than 1 indicate that the genotype is less responsive to changes in environmental conditions. These genotypes may perform assessed trait relatively well under poor conditions but do not show much higher values with better conditions. Genotypes with the lowest slope should be chosen because of their stability in adverse environments. The variation in response to the environment may indicate strategic genotype resource utilization.

The Finlay-Wilkinson regression slopes for all evaluated genotypes across the four traits are detailed in **Supplementary Table 5**, illustrating the range of genotypic reactions to environmental conditions. These results imply that breeders should carefully select genotypes based on the intended cultivation regime and location, which is consequential for choosing stable genotypes for parent selection. In summary, the Finlay-Wilkinson regression has yielded valuable insights into the stability and adaptability of genotypes within our trials, guiding selection criteria for future breeding programs that aim to develop cultivars tailored for broad adaptation or specific favourable environmental responses.

## Discussion

Altogether, 360 accessions from different regions of the world were evaluated in phenotyping trials. We found a significant variability in the observed agronomic traits. We were interested not only in the variability of the selected important characters, but especially in their stability in different conditions. The stability of performance of individual genotypes grown in different environmental conditions is important for soybean growers and breeders (Rani and Kumar, 2022).

The geographical origin of individual genotypes played a very important role in our dataset. The most represented were accessions from Europe, North America and China. China itself is a very important region from the point of view of genetic resources, because it represents the domestication centre of soybean (Kim et al., 2012). It is not surprising that cultivated soybean prefers warm and humid climatic conditions during the vegetation period, typical for the region where soybean was firstly domesticated. The best soybean producing areas are located in warm areas with sufficient rainfall during soybean vegetation period (Gonçalves et al., 2021).

Selecting the best varieties should be considered with other existing limitations such as irrigation and field management, soil, growing season and its climatic conditions. Differences in crop management and agricultural practices also contribute to variations in yield and biomass (Araji et al., 2018). Soybean grows optimally at temperatures between 20 and 30°C (de Avila et al., 2013). High temperatures during flowering can cause reduction in seed number and seed weight. If the high temperatures are associated with a drought, the losses of grain production are even higher. On the other hand, the regions with temperatures below 10°C are not suitable for soybean cultivation (de Avila et al., 2013).

Concerning the climatic conditions, there are several critical moments, influencing the successful soybean cultivation: 1) sufficient rainfall during the emergence period, 2) absence of ground frosts during the emergence period, 3) combination of warm weather with occasional rainfall during the growing season and 4) warm and dry weather during the ripening period. Additional irrigation in case of drought significantly helps to increase the soybean yields. The importance of irrigation was mentioned by Karges et al. (2022). In their trial irrigation increased soybean yields by 41% on average. In the year with sufficient precipitation, no additional irrigation is necessary (Karges et al., 2022). We observed differences between different maturity groups and growth types of soybeans in sensitivity to stress conditions. Early and short-growing cultivars are considered to be the most tolerant under mild and severe water stress (Araji et al., 2018).

Climatic conditions limit the expansion of new cultivation areas of soybean. The limiting factors are favourable temperatures and enough precipitation, mainly at the time of germination and flowering (Gawęda et al., 2020; Mandić et al., 2017). Soybean requires a sufficient number of warm days to mature. Kühling et al. (2018) reported a site in northwest Germany as sufficient for soybean cultivation. Rainfall during the later phase of the season, however, can have negative effects on the maturation process. Since soybean will be in the field until autumn in the northern parts of Europe, the risk of higher rainfall rates increases towards the harvest period. Soybean pods are fragile and repeated cycles of drying and wetting increase pod shattering and loss of seeds (Đorđević et al., 2021). Żarski et al. (2019) studied climatic risks for soybean cultivation in central Poland. Their analysis led to determination of the following unfavourable climatic conditions for soybean cultivation: shortening of the active growth period, a delay of the date on which the soil warms up to 8°C at a depth of 5 cm, occurrences of meteorological and agricultural droughts and of late spring ground frosts.

Soybean requires a soil temperature of 8 – 12°C for germination, with lower temperatures reducing plant density and yield (Yamaguchi et al., 2014). It is also sensitive to cold at flowering (Balko et al., 2014) with temperatures below 8°C associated with poor fertilization of ovules and subsequent blossom dropping (Yamaguchi et al., 2014). On the other hand, heat stress during the flowering stage can decrease yield significantly (Araji et al., 2018).

Future changes in temperature, rainfall, and CO_2_ concentration will influence soybean growth and the final grain yield. Soybean will achieve an optimal threshold temperature in the future, leading to yield increases in the 2030s in temperate climate areas (Araji et al., 2018). Thanks to climate change, soybean cultivation is moving to suboptimal conditions, increasingly to colder conditions, areas in high latitudes or in high altitudes. Those areas are for example southern Scandinavia or northern Canada. On the other hand, the plants have to deal with even greater fluctuations in the weather during the growing season.

Identification of early maturity genotypes enables the soybean growers and breeders to provide suitable genotypes for marginal areas, especially those with colder climate. Based on the results of our experiments, we can recommend for cultivation in marginal areas those varieties that matured successfully in both experimental years at locations in Germany and Belgium. For successful soybean cultivation in marginal areas, it is important to choose not only early but also photoperiod-insensitive cultivars. These cultivars seem to be more strongly influenced by temperature, with higher temperatures resulting in earlier flowering (Kurasch et al., 2017).

The earlier the soybean genotype is, the more it is positively influenced by the length of the day. Late genotypes are not so much affected by the length of the day and grow well even under the condition of shorter days (Yang et al., 2019). Later breeding efforts for long-day suitability gradually added three more groups at the lower end (0000–00; Jia et al., 2014). Even though this day length × temperature range covered by the MGs already allows a broad adaptation across Europe, the early MGs – especially 0000 – currently produce significantly lower yields (Ortel et al., 2020) and are, therefore, not very attractive. Season length, as a combination of both factors, is, therefore, still a major constraint for growing soybean in northern latitudes (Yang et al., 2019).

Soybean yield and seed protein content are still the main breeding goals (Berschneider, 2016). We identified ten most promising soybean genotypes through the analysis of their seed yield and protein content using BLUPs. Genotypes were ranked based on their performance in producing high seed yield and high protein content, providing valuable insights into potential candidates for further breeding or cultivation efforts.

Identification of the most stable genotype even under variable environmental conditions is very important for the future cultivation and breeding of soybean genotypes. Current breeding efforts have concentrated on developing more drought-tolerant varieties as well as on weed-suppressing traits (higher and more branched plants with more leaves), which are especially important for organic production systems (Klaiss et al., 2020). Nendel et al. (2023) studied soybean potential in Europe under the conditions of climatic change. Their projections suggest a substantial increase in potential soybean production area and productivity in Central Europe, while southern European production would become increasingly dependent on supplementary irrigation. While wet conditions at harvest and incidental cold spells are the current key challenges for extending soybean production, the models and climate data analysis anticipate that drought and heat will become the dominant limitations in the future. Breeding for heat-tolerant and water-efficient genotypes is needed to further improve soybean adaptation to changing climatic conditions (Klaiss et al., 2020).

The observed variability in phenotypic traits across diverse environments and genotypes underscores the imperative need for integrating genotype-environment (G × E) interaction analyses within breeding programs. The results of Finlay Wilkinson regression recognize genotypes that have stable trait response across environments (slope near 1) as well as those that show good performance in marginal conditions (slope different from 1). The differences in the environmental conditions of the trials, as a result of the extreme dry years and the additional irrigation, were so different that the slope of the genotypes for the seed yield trait has values far above of 1. Even so, it is possible to select from relative comparisons between each other those genotypes that are stable in their response to extremes of conditions and those that are solely dependent on good conditions. This approach is crucial for elucidating the differential responses of genotypes to varying environmental conditions, thereby facilitating the development of crops that exhibit enhanced resilience, adaptability, and consistent high-yield performance across a spectrum of climatic and agronomic scenarios.

Our investigation revealed significant fluctuations in seed yield attributable to varying environmental conditions, highlighting the pronounced effect of climatic factors such as temperature and precipitation on yield outcomes. The observed yield variability not only reflects the genetic diversity among soybean genotypes but also delineates a spectrum of resilience and yield potential under diverse conditions. Importantly, certain genotypes exhibited notable resistance and stable high-yield performance irrespective of environmental stressors. This phenomenon of yield fluctuation and the consequent G × E interactions, which highlight the differential genotype responses to environmental conditions, have been substantiated by studies from Alghamdi (2004), Krisnawati and Adie (2018), Oliveira et al. (2006), and Ngalamu et al. (2013). These studies underscore the significance of selecting genotypes that maintain stable performance across varied environments, contributing significantly to the predictability and reliability of crop yields. Additionally, Elmerich et al. (2023) identified specific eco-climatic factors influencing yield in early maturity soybeans, further emphasizing the importance of stability in performance.

The variation in plant length across different environments and genotypes underscores a complex interplay between soybean genetics and environmental cues, significantly impacting plant structure and consequently, plant density, harvesting efficiency. The importance of stable performance in terms of plant architecture, facilitated by the resilience of specific genotypes against environmental fluctuations, is critical for optimizing agricultural output. The contribution of environmental factors to G × E interaction, particularly in defining plant traits, was explored by Kang et al. (1989), who highlighted the critical role of weather variables and rainfall in influencing plant architecture. Furthermore, Yang et al. (2020) extended this understanding by identifying significant G × E effects on traits related to plant height and pinpointing specific quantitative trait loci that contribute to plant height across various environmental settings, while in soybean the results of mapping studies can be further specified to the level of causal mutations using synthetic phenotype association (Škrabišová et al., 2022).

Plant length is closely related to stem determination. Stem determinacy is quite variable among evaluated genotypes (Borra-Serrano et al., 2020). An important aspect for breeders and breeders is also the difference in sensitivity between determinate and indeterminate soybean genotypes. Indeterminate varieties are least affected by day length. Determinate varieties perform less well at high latitudes (Kato et al., 2019) including a large part of Northern and Western Europe (Schori et al., 2003).

Additionally, although there is observed variability in the timing of maturity (R8 stage), the response of different genotypes to environmental conditions demonstrates remarkable stability. This common stability facilitates the adaptation of genotypes to the photoperiod and temperature conditions characteristic of new geographic regions. The research conducted by Persa et al. (2022); Kantolic and Slafer (2005) and Elmerich et al. (2023) collectively highlights the significant influence of photoperiod on the development and yield of soybeans.

Regarding the nutritional quality of soybean seeds, specifically protein content, results indicate minimal response variability among genotypes across different environments, suggesting an inherent stability in nutritional quality despite environmental challenges. This stability offers promising prospects for enhancing the nutritional value of soybean crops through the identification and selection of genotypes that consistently exhibit high protein content alongside stable yield performance. Studies by Perić et al. (2021); Natarajan et al. (2016); Carrera et al. (2014) and Sethi et al. (2012) further confirm the feasibility of identifying genotypes with superior protein content, despite environmental variations.

At the end of this article, it is good to mention the main practical outputs of our study for breeders and soybean growers. On the basis of the above-described and then discussed results, it is possible to select, on the one hand, genotypes that have both a high yield and a high protein content, regardless of the growing conditions (Berschneider, 2016). These genotypes are interesting not only for breeders, as input materials for the breeding process, but also for soybean growers (Singh and Shivakumar, 2010). The second important group consists of mainly early genotypes, which are interesting from the point of view of cultivation and breeding for marginal conditions (Singh and Shivakumar, 2010). In our experiments, the riskiest factors from the point of view of soybean cultivation appears to be a period of long and intense drought, especially in the spring period, when soybeans go through a period of intensive growth and development (Klaiss et al., 2020; Saleem et al., 2021). For this reason, for the effective cultivation of soybeans, it is necessary to have an irrigation system available, without irrigation it can lead to a very significant reduction in yield (Karges et al., 2022).

## Conclusions

Within the EUCLEG project, 360 accessions from different regions of the world were evaluated in phenotyping trials using the same methodology. The five most important soybean traits were selected for detailed analyses: plant emergence, plant length, protein content, seed yield and R8 stage. We found a significant variability in the observed agronomic traits. From the point of view of soybean breeding and cultivation, however, the most important characteristics are seed yield and protein content. The ten most promising genotypes from the point of view of protein content and yield were selected and could be used in breeding programmes. Our results and observed variability in phenotypic traits across diverse environments and genotypes underscore the need for integrating genotype-environment interaction analyses within breeding programs.

## Data Availability

The original contributions presented in the study are included in the article/**Supplementary Material**. Further inquiries can be directed to the corresponding author.
