# Supplementary material for: Phenotypic characterization of soybean genetic resources at multiple locations: breeding implications for enhancing environmental resilience, yield and protein content
Source: Front Plant Sci. 2025 Apr 7;16:1422162. doi: 10.3389/fpls.2025.1422162 (PMC12009817; doi:10.3389/fpls.2025.1422162)
Supplement: Supplementary file 2 [file Table2.docx]

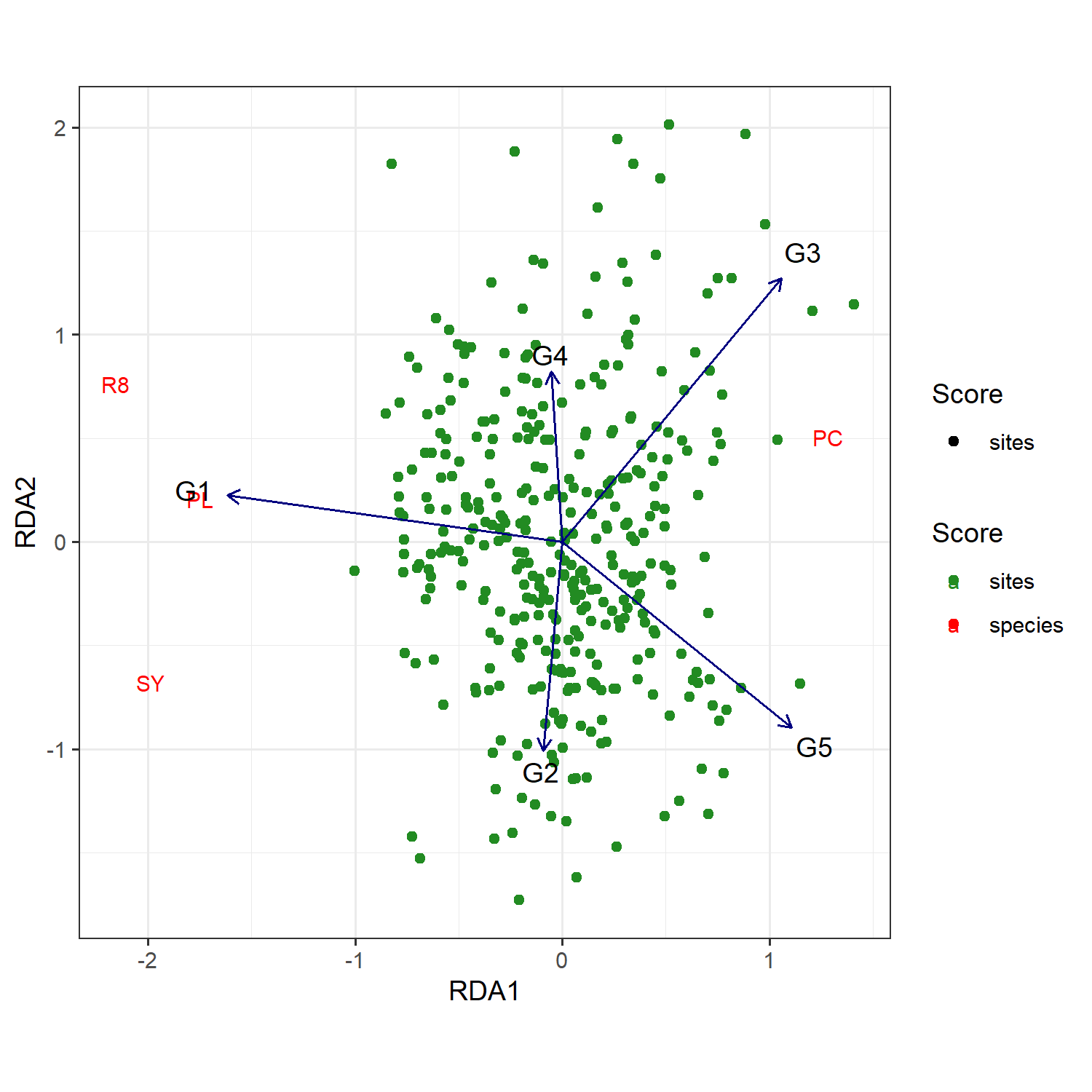


Supplementary figure 1: Biplot of RDA scaling 2 analysis showing associations between accessions (green points) and genetic clusters (arrows) with position of the key traits R8 stage (R8), plant length (PL), protein content (PC), seed yield (SY).
